# Psychiatric spaces: a phenomenological case study of staff perspectives after relocation to a new mental health facility

**DOI:** 10.1080/17482631.2025.2485697

**Published:** 2025-04-01

**Authors:** Anne Hagerup, Carina Ribe Fernee, Helle Wijk, Göran Lindahl, Sepideh Olausson

**Affiliations:** a Institute of Health and Care Sciences, Sahlgrenska Academy, University of Gothenburg, Gothenburg, Sweden; b Inland School of Business and Social Sciences, Inland Norway University of Applied Sciences, Lillehammer, Norway; c Department of Child and Adolescent Mental Health, Sørlandet hospital, Kristiansand, Norway; d Department of Quality strategies, Region Västra Götaland, Sahlgrenska University Hospital, Gothenburg, Sweden; e Department of Architecture and Civil Engineering, Chalmers University of Technology, Gothenburg, Sweden; f Division of Construction Management, Department of Architecture and Civil Engineering, Chalmers University of Technology, Gothenburg, Sweden; g Department of Anesthesiology and Intensive Care/Sahlgrenska, Sahlgrenska University Hospital, Gothenburg, Sweden

**Keywords:** Affordances, mental health facility, nature, phenomenology, physical environment, psychiatric staff

## Abstract

**Introduction:**

Patients in mental health care rely on staff for their well-being, security, and quality of treatment. However, staff’s perspective of the physical environment where care takes place remains underexplored. Their insights are crucial to understanding how the environment impacts the quality of care. Therefore, the aim of this study is to explore the meanings of the physical environment for inpatient care according to staff shortly after relocation to a new mental health facility.

**Methods:**

The study employed a phenomenological approach and focus group interviews with 20 staff working in a newly built mental health facility. Data were analysed using van Manen’s existentials and guided by the theory of affordances.

**Results:**

The primary findings were as follows: (a) attempting to provide a therapeutic atmosphere, (b) design as symbolism, (c) altering the physical environment means altering time, (d) offering spaces for connection and communication, and (e) embodying the new mental health facility.

**Conclusion:**

The findings indicate that regardless of whether affordances are actualized, opportunities and obstacles in the hospital environment impact the staff’s ability to provide inpatient care according to their standards. Conflict arose due to obstacles inherent in the organization and structure of the new mental health facility that limited opportunities to utilize possible affordances.

## Introduction

Throughout history, psychiatric inpatient treatment has often occurred in secluded buildings, symbolizing the confinement and separation of “madness.” These practices and spaces directly or indirectly exerted power over marginalized patients and reflect an outdated approach to mental health care, supporting Foucault’s (2003) critique of architecture as a tool used to control those with mental illness. For instance, inadequate maintenance efforts and buildings “inherited” from somatic use remain issues associated with mental health facilities, suggesting that psychiatric care is not a priority of health care (Lundin, 2021). The primary goal of health services is to offer humanistic and evidence-based treatment to people in need, whether their ailment is of physical or mental origin. However, within the field of mental health care, it may be difficult to support a diverse patient population’s multitude of needs, often including a combination of diagnoses, sensory processing difficulties, and cognitive impairments (Shepley & Sachs, 2020; Tan et al., 2018; Weber et al., 2022). Purpose-built environments and appropriately designed spaces can support the provision and quality of care (Rodríguez-Labajos et al., 2024). However, there is a lack of research exploring the various ways the physical environment of mental health facilities may support and improve inpatient care (Weber et al., 2022). Therefore, the focus of this study is exploring the meanings and impact of the physical environment on staff’s everyday caring practices in a new purpose-built mental health facility.

## Background

An expanded view of health care involves the consideration and intentional use of the supportive mechanisms that may be found in the immediate physical surroundings of places for care, including nature, recognizing that health care is not provided in one specific setting or in one-dimensional contexts (Battisto & Wilhelm, 2020). Carefully designed health care buildings, including biophilic design and access to nature, could have the potential to support mental well-being and therapeutic work in future-oriented health services.

Attention to physical environmental factors in health care can increase user satisfaction and ensure that places of care are designed to reduce environmental stressors (Iyendo et al., 2016; Ulrich et al., 2008) and offer supportive spaces (Jutzi et al., 2024). Shepley et al. (2013, 2016) identified influential features in the physical environment that appear to have a positive impact on patients and staff in psychiatric settings. These include nature connectedness, positive distractions, autonomy and spontaneity, the provision of indoor/outdoor therapy, and staff support. A well-maintained environment may improve staff mood, reduce staff absences, and support treatment. Furthermore, access to nature appears to provide psychological, physiological, and cognitive benefits (Tan et al., 2018). Tan et al. (2018) further emphasized that natural features, such as plants and gardens, are valued by staff and can have a positive impact on job satisfaction. Research has shown that human health and well-being are positively affected by exposure to nature, where the availability of gardens and natural scenery can be relevant to mental health care by providing mental restoration (Abbasian et al., 2020; Connellan et al., 2013; Rehn-Groenendijk et al., 2022). The natural environment has to be perceived positively for it to offer health benefits, the theory being that our oxytocinergic system plays an essential role in mediating the mental and physiological effects of contact with nature (Grahn et al., 2021). Oxytocin is believed to enhance social interaction, reduce levels of fear and stress, and increase levels of trust and well-being (Grahn et al., 2021).

This study is part of a larger project following a newly built mental health facility in Norway from the planning stage through to the relocation of staff and patients to the new building. This new mental health facility was designed to provide a supportive environment for inpatient care and to incorporate nature elements in the immediate surroundings. In a previous study, we found that there was an explicit awareness and knowledge of the importance of a building being *future-oriented*, in terms of enabling novel treatment approaches (Hagerup et al., 2024). The present study investigates how this purposefully designed mental health facility supports everyday care practices from the perspectives of the staff. Drawing on the theory of affordances (Gibson, 1979; Jiang et al., 2024), we aim to explore the meanings of the physical environment for inpatient care according to staff shortly after relocation to the new mental health facility.

The research questions that guided this study are as follows:

1. How do staff experience and engage with the physical environment in their daily clinical practice in the new mental health facility?
2. What meanings emerge from the experiences of everyday caring in the new facility?

## Materials and methods

### Theoretical standpoints

This study employed the theory of affordances (Gibson, 1979), a perspective within environmental psychology. The theory of affordances focuses on the interplay between individuals and their surroundings, aiming to understand how the thoughts, feelings, and behaviours of a person are influenced by their environment. Furthermore, it is concerned with both opportunities and hindrances that might exist in a given environment (Schultz & McCunn, 2022). Environmental psychology at its core focuses on place and space, arguing that social interaction does not occur in a vacuum—individuals are always *somewhere*, and their immediate surroundings affect their well-being (van der Linden, 2019). The application of an affordances perspective in the design and research of supportive environments provides a shared language and a common theoretical basis upon which to explore the connection between the initial intentions of a design, its artefacts, and its subsequent use (Maier & Fadel, 2009).

### Study design

This exploratory case study used a qualitative phenomenological approach (van Manen, 2014). In the context of environmental psychology, qualitative research can generate in-depth knowledge and yield insights into clinical environments through participants’ lived experiences (Lloyd & Gifford, 2024; Ratcliffe et al., 2024). Semi-structured focus group interviews (Wibeck, 2010) with psychiatric staff (nurses, social workers, occupational therapists, various environmental therapists, and psychologists) were conducted to explore how they experienced the new hospital environment in relation to their daily clinical work. Furthermore, insights into the impact and meanings of the physical environment for inpatient care were explored through these focus group interviews (Wibeck, 2010).

Phenomenological inquiries are concerned with the study of lived experiences, where the concept of lifeworld is essential and refers to the subjective, taken-for-granted everyday world as it is lived and experienced immediately (Husserl, 2001a, 2001b; Moran, 2012). This study adopted a phenomenological lens because our aim was to understand the participants’ everyday clinical work in the new hospital setting and how the environment shaped their caring practice. Intentionality is a central concept in phenomenology and means that a phenomenon is always understood as something meaningful for our consciousness (Husserl, 2001a, 2001b; Moran, 2012). From the phenomenological lens, a place is not an empty container but a meaningful background to everyday life. Place, time, and body exist on a continuum, as humans always exist in a place against a temporal horizon. Thus, our intention was not to explore the lived experiences of therapeutic practices alone but in relation to the meanings of the environment as a “place for care.” Thus, the environment had to become figural and be positioned at the forefront of this study for its meanings to be explored. Consequently, we drew the staff’s attention specifically and deliberately to how they experienced the physical environment in the new facility and its meaning and impact on their day-to-day caring

### Setting—the case

Case studies, unlike other research methods employed for data collection or analysis, are characterized as specific units for analysis (Willig, 2013; Yin, 2018). In this study, the case chosen was a new mental health facility that was developed with a specific focus on nature and supportive building design. The mental health facility is part of a public hospital located in Southern Norway that provides specialist health care. At the time of data collection, the site of inpatient mental health care had recently been relocated from old and outdated premises to this modern and future-oriented mental health facility on the outskirts of the hospital grounds on a hill immediately bordering the forest.

A key feature of this brand-new and intentionally designed mental health facility is that it offers the possibility of utilizing the surrounding nature for therapeutic ends (Hagerup et al., 2024). All patient rooms and common areas have ground-level access to, and views of, green areas. The facility was completed in the spring of 2023. It provides inpatient care through seven units for adult patients and a unit for adolescents aged 12–18 years. The eight units each have 10 patient rooms and are all locked wards. The units are as follows: (1) the Psychiatric Emergency Unit; (2) the Psychiatric Subacute Intensive Unit; (3) the Unit for Assessment of Psychotic Disorders; (4) the Unit for Psychosis and Addiction Disorders; (5) the Reinforced Unit for Psychosis and Addiction Disorders; (6) the Forensic Unit; (7) the Unit For Geriatric Psychiatry and Cognitive Impairment; And (8) the Child and Adolescent Unit. All inpatient units are situated on the ground level of one building to allow easy access to nature, making the indoor—outdoor flow seamless. Patients can access the outdoors by entering the atrium gardens when they wish; they do not have to ask for permission or be accompanied by staff. Housing all units in one building also facilitates staff cooperation among units, contributing to increased security. Please see Photographs 1, 2 and 3.

**Photograph 1.**
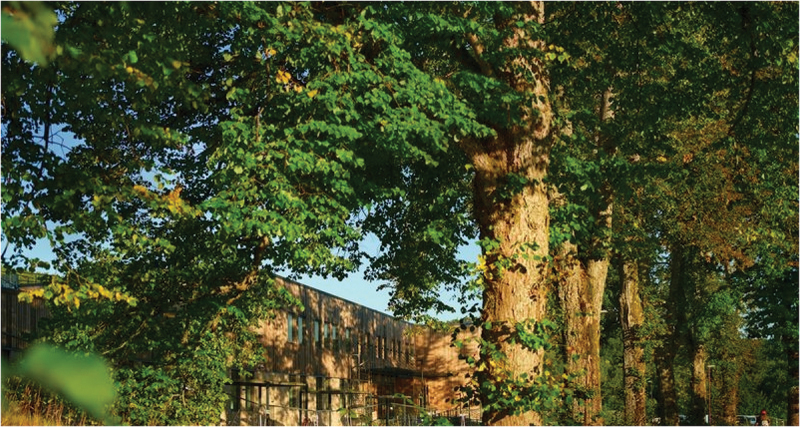
The main entrance to the building surrounded by nature.

**Photograph 2.**
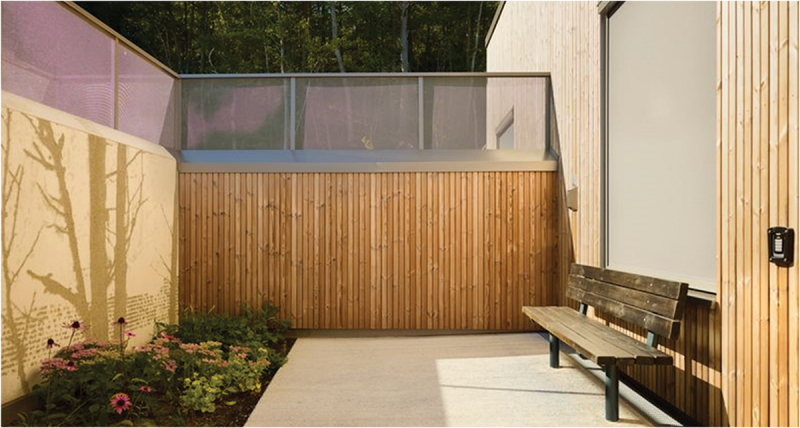
Private garden of the isolation room.

**Photograph 3.**
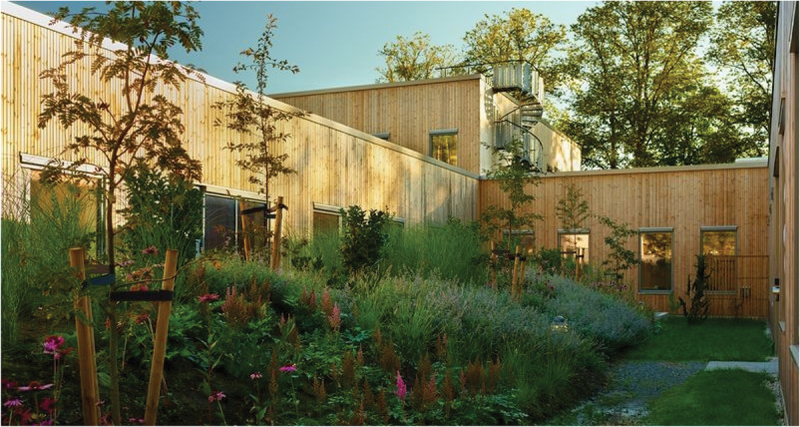
Quiet area outside the patient rooms.

### Recruitment and participants

To capture staff members’ lived experiences, we estimated that three focus group interviews, each including at least one staff member from each of the seven adult inpatient units, would provide meaningful insights for our phenomenological study. Participant recruitment was organized through information meetings with unit leaders. Based on the information meetings, the leaders recruited three staff members each by means of purposeful sampling (Polit, 2022). Unit leaders were informed that participation would be based on voluntary informed consent and that participant representativeness in terms of professional background (i.e., nurse, psychologist, occupational therapist, and other specializations), gender, age, and years of practice would be preferable.

All participants had received information about the study from their unit leaders, along with written information about the purpose of the study and time when the three focus group interviews would occur. Participants signed and submitted their consent forms directly to the principal researchers (AH and CRF) when they met up for the focus group interviews. The focus groups included one staff member from each of the seven units for adult patients, with the exception of the second focus group, which was missing a staff member from Unit Four. Two focus groups included seven staff and one group included six, meaning we had a total of 20 participants (*n* = 20). This was considered a representative sample for the purpose of a phenomenological case study. The participants were between 23 and 57 years of age, with an average age of 41 years. Most of them had worked in the old buildings prior to the relocation. The interviews were carried out in August 2023, only a few months after the March—April relocation of patients and staff to the new mental health facility. Participant characteristics are shown in Table I.

**Table I.** Participant characteristics across the three focus groups.

Participant number	Gender	Age	Professional background	Hospital unit	Years of practice	Interview duration
Focus Group A						56 m 41 s
1	M	25–34	Nurse	1	6	
2	M	25–34	Nurse	5	1	
3	M	35–44	Nurse	2	9	
4	M	25–34	Nurse	3	3	
5	F	35–44	Psychologist with specialization	6	13	
6	M	45–54	Psychiatric nurse	4	17	
7	F	55–64	Occupational therapist	7	6	
Focus Group B						52 m 20 s
8	M	45–54	Environmental therapist	6	23	
9	F	55–64	Psychiatric nurse	3	30	
10	F	25–34	Psychiatric nurse	1	9	
11	F	45–54	Nurse	5	25	
12	F	45–54	Psychiatric nurse	2	21	
13	M	35–44	Psychologist	7	7	
Focus Group C						52 m 51 s
14	F	25–34	Social worker	4	11	
15	F	45–54	Nurse	7	21	
16	F	35–44	Nurse	5	11	
17	F	25–34	Sociologist	6	10	
18	F	25–34	Nurse	1	5	
19	M	45–54	Social worker	3	29	
20	M	35–44	Psychiatric nurse	2	18	
Mean	9 male/ 11 female	40.75			13.75	53 m 37 s

### Data collection

In each focus group interview, the researchers posed open-ended questions to invite the participants to talk about their experiences of the physical environment and its impact on their everyday work. The semi-structured interview guide contained the following questions, among others:

- How does the new hospital environment and its surroundings impact your daily work with patients?
- What does the new building and environment mean for your everyday practice?
- What opportunities and/or obstacles do you see in the environment in relation to your patient care?

The researchers also asked follow-up questions to elicit examples and to explore the participants’ responses and reflections further. All interviews were conducted in a meeting room in the new mental health facility, with the first author acting as moderator and the second author as facilitator (Wibeck, 2010). The interviews lasted between 52 and 56 minutes, as the participants were only allowed an hour away from their respective units. The interviews were audio recorded and transcribed verbatim by the first author.

### Data analysis method

The focus group interviews were analysed using the hermeneutical phenomenological method outlined by van Manen (1997), where the meanings of the physical environment of the new mental health facility were explored and reflected on through the staff’s perspectives. The analysis followed *Guided Existential Inquiry* (van Manen, 2014), which outlines five fundamental existentials: (1) relationality—lived self-others; (2) corporality—lived body; (3) spatiality—lived space; (4) temporality—lived time; and (5) materiality—lived things. Together, these existentials allow for multidimensional phenomenological exploration.

van Manen’s (2014) reflective inquiry process consists of six steps: (1) Formulate a research question based on the nature of lived experience; (2) Capture the phenomena (lived experiences) through focus group interviews; (3) Explore the overall meanings participants ascribe to their experiences; (4) Write and rewrite with the intention of describing the phenomena and revealing the participants’ feelings, thoughts and attitudes; (5) Focus on the research questions while simultaneously maintaining a strong connection to the phenomena; and (6) Consider the parts and the whole by constantly balancing the context of the research.

We carried out the analysis by engaging with the text through repeated readings of the transcribed material to uncover layers of meaning related to van Manen’s (2014) five existentials. These existentials served as guiding lenses rather than fixed categories. In using a reflective approach, meanings, words, and sentences related to the aim of the study and the research questions were identified. Relevant meanings were noted throughout the reading process. Thereafter, based on an open mindset, similar aspects of experience were grouped together, and writing itself became a fundamental part of the analysis—allowing the phenomena to be voiced and described more deeply.

The process was rooted in dialectical movement involving continuous interpretation, reflection, and refinement, ensuring that each existential dimension was contextually grounded in the lived experiences of the participants. Lastly, all five existentials were further developed and presented as narrative descriptions, incorporating participants’ voices and phenomenological reflections to stay true to the depth and complexity of the phenomena.

### Rigor

To ensure the scientific rigour and quality of this study, the authors adopted a phenomenological attitude, which meant embracing a genuine ambition to understand the phenomena in new ways. Nevertheless, as preunderstandings may have influenced the analysis (Kvale & Brinkmann, 2019; Silverman, 2017), we systematically challenged our own preunderstandings through openness and critical discussions throughout the entire research process. The authors’ different perspectives and experiences are important to quality assurance in phenomenological practices.

The first (AH) and second (CRF) authors carried out the interviews in this study. AH analysed the results in close collaboration with SO and based on continuous feedback from the research team. AH is a PhD student, a clinical psychologist specializing in adult psychology, and an environmental psychologist. CRF is a senior researcher specializing in clinical research and the implementation of outdoor therapy. HW is a professor and a nurse. GL is a professor and an architect. Both HW and GL have expertise in research on health care environments. Finally, SO is an associate professor with extensive experience in both qualitative research and research in the area of health care environments.

### Ethical considerations

This study was conducted in accordance with the Declaration of Helsinki and ethical principles for medical research involving human subjects. The participants were informed that they had the right to withdraw from the study at any point without explanation or detriment. This was done both orally and in writing. Participants were given time to consider the invitation and ask questions, in addition to information about the study. Potential participants were also given contact information for the responsible researchers and institutions. All participants gave written consent to participate in the study. This study was approved by the Norwegian Agency for Shared Services in Education and Research and the Swedish Ethical Review Authority.

## Findings

Based on a phenomenological analysis of three focus group interviews with a total of 20 psychiatric staff members, the findings are presented with respect to the five overarching existentials outlined by van Manen (2014): (a) lived space, (b) lived things/objects, (c) lived time, (d) lived relationality, and (e) lived body. We further identified sub-existentials within each overarching existential to capture the meanings ascribed to lived experiences from the perspectives of psychiatric staff.

### Lived space (spatiality): attempting to provide a therapeutic atmosphere

The existential theme of spatiality is concerned with how the different dimensions of the new mental health facility and its surrounding environment might shape therapeutic practices in everyday clinical work and each individual’s being and actions. This theme also scrutinizes the relationship between physical space and a place for care, shedding light on two different worlds: the inner dimensions of a person’s psyche and outer dimensions of a physical space as a place of care.

#### Enabling a space for care

The participants’ narratives revealed how the spatial design of the new facility and its integration with nature influenced everyday therapeutic practice. The physical environment itself was described as offering a range of stimulating features, such as large windows that let in bright daylight, high ceilings that create a feeling of spaciousness, and the choice of soothing colours throughout. Closeness to nature entailed a desire *to be* in nature. However, the organization of staff and work tasks conflicted with the affordances of the physical environment and its purposes. This meant that despite all the affordances on offer, organizational barriers prevented the new mental health facility from fulfilling all its promises (i.e., realizing the spatial affordances of a place for care).

However, the spatial arrangement fostered a sense of meaningfulness and allowed the participants to engage with the physical environment in a way that felt supportive and essential to them in their everyday therapeutic practice: “And it is clear that the building itself probably makes the treatment situation better” (FG 1, P6). In particular, the soundscape and visibility of the new environment were emphasized.

#### The sound of silence

The sound environment of the new building was experienced as a positive quality. Little noise filters from room to room, encouraging therapeutic benefits and comfort. For example, the patients found the lack of noise and residual sounds to help them sleep, which was seen as an important prerequisite for benefiting from therapy: “If the patients do not sleep well, it is difficult to treat them [the patients] the next day” (FG 2, P1). However, the silence also created feelings of insecurity because the soundproofing meant that staff could not pick up on auditory cues and they perceived there to be a risk of escalating situations going unnoticed. The participants voiced a fear that they might fail to pick up on noise or sounds from the patient areas if they are in the staff area: “Now we can sit in the staff room and hardly notice that anything is happening outside because it is so soundproof” (FG 3, P3). In addition, if a staff member needs help, it would be more difficult to alert other staff; the staff further voiced a fear of being more vulnerable in an emergency: “Whereas now, you can be there alone until it happens because no one picks up what’s going on around you in a way” (FG 3, P5).

#### Visibility for good and bad

The built environment was designed to provide overviews and lines of sight, which means total visibility in the common areas, such as the living room, halls, and kitchen of each hospital unit. The participants described how the lines of sight provided security and safety by providing overview, in addition to enhancing predictability regarding what might be happening in the environment. Lines of sight also affect the density of people; the corridors are wide and spacious, providing a natural flexibility. Increased lines of sight contributed to the feeling that the hallways were less crowded, making it seem as though there was a lower density of people. However, the participants observed that this very visibility could create feelings of frustration, as it meant being unable to find a safe space or place to be alone, outside of the patients’ individual rooms. The openness and constant visibility evoked the feeling that everybody was in the “same” room all the time, leaving no space for seclusion: “We are in a way together absolutely all the time” (FG 2, P3).

In contrast, the open floor plans and modular furniture made the participants experience these arrangements as facilitating activities and interactions. Staff reflected on the duality of the patients’ experiences of the environment. While some patients experienced the environment as facilitating feelings of safety, others experienced the opposite—these individuals felt they were under constant surveillance.

Because before there was a corridor and a TV room all by itself. We couldn’t see into the TV room before, so things could happen there that we didn’t catch. But now we have an overview of all directions, so we see what is happening. In that sense, it is safer for patients in terms of security. (FG 2, P 4)

#### Absence of intended affordances

The participants noted that the building and its design at times prevented them from offering activities for patients in the reinforced or forensic units, to the point where this patient group had limited opportunities. For example, access to outdoor activities or the in-house gym was restricted because these patients were not allowed to go through the common public areas on their own. Therefore, they were prevented from fully benefiting from the psychological and physical effects of exercise and moving freely, unless a sufficient number of staff were available to accompany them: “The patients are frustrated because they want to go out; they don’t understand that it is not possible to just go for a short walk to the gym that is there” (FG 3, P7). However, in the evenings, the public area was closed, making access more secure and available to all patients.

Participants reflected on how nature and the use of outdoor spaces could have an impact on the therapeutic process. Nature felt both close and far away, meaning that it was experienced as harder to reach from the new facility, as there were more barriers to access than in the old building where nature was just outside. The participants also reflected that the atrium gardens of the new building could counteract the need to go outside into nature because the gardens are more accessible: “There may have been less demand at the start [right after relocating] for hikes and the like when they can get some fresh air in the atrium” (FG 1, P2). However, the atrium was not believed to offer the same potential benefits as a prolonged period in wild nature or, for example, going on a hike.

### Lived things (materiality): design as symbolism

This existential theme concerns the materiality of the environment and how things or objects shaped staff experiences of the place and their therapeutic practices. Objects contribute to the identity of a place and shape our expectations of an environment.

#### Altering atmospheres

The participants felt that the design and materials used in the facility helped to strengthen the patients’ sense of comfort and safety. Staff actively used the opportunities the design and materials offered, making a conscious choice to attract the patients’ attention to the many possibilities to be found in the environment, including taking gradual steps towards normalcy, meaningfulness, and hope:

I think it’s a boost to bring in the light and be able to persuade them to open the blinds to look out. And then talk about whether they’re trying to divert their attention from the bed and straight into something beautiful. (FG 1, P7)

Being able to regulate the indoor lightning was described as providing an opportunity to offer different atmospheric states and ambiences, as well as to symbolically represent the rhythm of the day or an experience. For example, dimmed, warm light late in the evenings and at night signalled less activity, coziness, rest, and preparation for sleep, and brighter light in the daytime meant getting out of bed, becoming energized, and preparing to engage in therapeutic activities.

#### Symbolizing dignity and professionalism

The participants spoke of “the unspoken that resides in the building”, referring to the material quality of the new facility. The building radiates a warm and welcoming atmosphere, which evolved into a sense of pride among staff and a sense of dignity among patients. The building resembles a hotel, and the design was understood to express a sense of care by the use of soothing colours and “warm inviting materials” that was conveyed to the patients though the building itself. Staff also felt that the new building signalled safety and security for patients by the buildings supportive design, conveying the idea that here, you will receive the best of care. A participant taking on the perspective of a patient arriving in the building stated, “I think I would have been a little safer because here, we would give you good treatment, when there is something [a building with a supportive design] new” (FG 2, P1). However, despite the quality of the building, a there is no design that can meet all patient needs.

#### One size fits all?

The participants experienced the design of the units as identical for all patient groups, despite their differing needs. For example, patients could remain in the forensic unit for several years. Staff expressed concern about the lack of opportunity to offer them ordinary experiences and physical objects, such as a wardrobe with enough space for all their clothes and sufficient storage space. In the unit for elderly patients with cognitive impairment, staff experienced difficulty reconciling the modern design with the elderly patients’ perceptions of what was familiar and felt to be safe. As such, staff had to remain close by to help:

In relation to the modern [design] and elderly patients, they do not go together. So we’ve had many challenges with that, just turning on the lights, and the light turning off in the bathroom when they sit on the toilet. They howl and scream. So, they don’t get it, and they’re anxious, and they become scared. (FG 3, P2)

### Lived time (temporality): altering the physical environment means altering time

Temporality is an existential theme that deals with how time is experienced in relation to phenomena under study. In this case, the analysis focused on how staff experienced the differences between subjective and objective time, meaning phenomenological time and clock time, and how the perceptions of time may have been altered when relocating to the new building.

#### Not having time to reflect and adapt

Time seemed to flow differently in the new facility in comparison to the old buildings. The layout of the building combined with a reorganization of minor tasks led to a feeling that there was not sufficient time for treatment/patient contact. This was an unforeseen result of organizational and spatial changes. The staff felt they frequently had to deny patients’ requests because they had to attend a meeting or complete practical tasks: “Then we have to take care of those who scream and shout the loudest. And those who might need a walk outside, who are depressed, they…, sorry, they don’t get the help they need” (FG 2, P5). The experience of a shortage of staff meant that they could only attend to urgent needs, leaving little or no time for reflection and adaptation.

#### Frustration over time-consuming tasks

The organizational structure of the building was believed to steal time from patient care. The staff felt inundated with numerous small, time-consuming tasks requiring their attention. There were designated staff for kitchen duties in the old hospital; in the new mental health facility, the staff were assigned these duties in addition to all of their other tasks: “Storing food, it takes a lot of time. Before the weekends, there is lots of food arriving. I’d rather go for a walk with the patients, right?” (FG 2, P4). These additional tasks, combined with a shortage of staff, created a feeling of pressure or urgency that limited the time and capacity (whether mental or physical) available for patient care, which is an essential element in therapy.

### Lived self-other (relationality): offering spaces for connection and communication between staff and patients

Relationality explores how oneself and others are experienced relative to phenomena under study. It delves deeply into the intricate ways in which the “self” and others are experienced and the ways in which subject—object relations are constituted.

#### Regulating the physical distance means regulating the therapeutic relationship

The participants described how the environment allowed adjustments in physical distance and proximity to patients, providing a greater sense of flexibility for accommodating the diverse needs of patients. The environment was perceived to play a role in fostering a relationship with the patients that could potentially increase their receptiveness to treatment: “It’s like that here too; if you get a bit of space, if you get something to fill your everyday life with, have someone to talk to, good food, it means a whole lot for treatment” (FG 2, P1). The participants emphasized that therapy is fundamentally a relational experience, even though the patients themselves may or may not be aware of it.

#### Relating to nature improves interaction

The participants noted that the staff—patient relationship changed in pace and focus when they were in natural surroundings. Conversations held outdoors were felt to differ from those that took place inside the facility: “But when we go and talk outside, we have much better conversations than when we talk about this inside” (FG 2, P1). It appeared that being outdoors with patients made it easier to foster trust and openness, creating a safer space in which to discuss difficult issues. One staff member experienced nature as stimulating the use of all the different senses and that this could calm the patients and make it easier for them to talk about their difficulties. The peaceful surroundings were seen as helping to construct a therapeutic relationship with patients, supporting them so that they became more receptive to therapeutic conversations. Staff also appreciated the opportunity to spend time in nature and the chance to clear their minds: “So, I think it [being outdoors] is better for me as well, that you can clear your head a bit” (FG 2, P6).

### Lived body (corporality): embodying the new mental health facility

This existential theme focuses on embodiment and how the body was experienced in this space and place for care. Taking care of patients involves physical work, reflecting the participants’ corporeal existence in the mental health care setting. It prompts examinations of how the body is perceived, experienced, and represented in shaping individual and collective identities, including the inscription of meanings onto bodies.

#### The vulnerable bodies

The participants described a change in the organization of the physical environment that influenced their physical responses and their performance. Some participants experienced the new surroundings as very positive, to a point where they felt more at ease and calm in their own bodies: “Personally, I noticed quite early on that I was less tired and overwhelmed during a working day” (FG 1, P6). They felt that the work environment was better in terms of increased access to daylight and better air quality compared to the old building. However, they also spoke about how the lack of staff rooms, although beneficial for the patients, could mean increased tension, tiredness, and stress among the staff as their everyday work became more demanding. They felt more vulnerable to work overload and fatigue because of the limited possibilities to retire to a more secluded place that offers silence and the chance to pause for a moment: “It’s tiring. We get very tired. We never have the opportunity to pull away and talk to someone privately. We miss that very much” (FG 2, P5). At times, there can be a lot of commotion in some of the units, which was perceived as demanding over longer periods. Therefore, the participants considered it essential for the staff to be able to retreat to a quiet, safe place to rest, debrief, and reset their minds and bodies.

## Discussion

This study explored the meanings of the physical environment and its significance for the everyday practice of inpatient mental health care. The setting was a new purpose-built mental health facility in Norway to which staff and patients had recently been relocated. Our findings reveal dilemmas that arose in the conflict between the availability of and barriers to making use of affordances. The new environment offered numerous opportunities that could be leveraged in therapeutic practice and everyday inpatient care. However, as the staff pointed out, organizational hindrances limited the use of these identified affordances.

A previous study (Hagerup et al., 2024) of the same case in Norway emphasized the importance of designing supportive environments in mental health facilities that are capable of adapting to future needs and enable novel treatment methods. The overall expectation in this study was that the physical environment of the new facility would support patients’ well-being and staff’s therapeutic practices. Supportive design is understood to mean the careful planning of a space with a warm and welcoming atmosphere that facilitates positive relationships and communication, enabled by a safe and calming environment where people feel at ease. Bearing this in mind, the current study found that the design of the new mental health facility only partially succeeded in realizing the intentions and aspirations expressed during the planning and design stages.

Drawing on the theory of affordances (Gibson, 1979), we explored the possibilities that the staff noticed the new environment offered as a “place for care,” both in the building itself and in the immediate surroundings. Kyttä (2004) differentiated between possible and actualized affordances, where a so-called potential affordance refers to a given opportunity within an environment or object. However, an affordance is only actualized when such a possibility is acted upon. If a possible affordance is not known, it cannot be consciously realized. However, frustration can occur if a possible affordance is known but there are hindrances to its being made use of or if a given environment affords no possibility of accommodating identified needs. In the current study, we found examples of both scenarios, which may prove relevant to other projects involving the careful design of buildings for the purpose of providing mental health care.

The process of relocation from the old buildings to the new mental health facility did not only include a physical transition but also significant organizational change in terms of the provision of care. Changes included a reduction in staff numbers and an increase in workload. A literature review on burnout and mental exhaustion among health care staff showed that these issues have garnered increasing focus, and the design of facilities for health care has been shown to significantly reduce and prevent the mental exhaustion of health care professionals by removing environmental stressors and providing restorative experiences (Jin et al., 2023). In this study, the analysis frequently revealed that for the participants, denying patient requests and only being able to respond to acute needs resulted in stress and dissatisfaction, as they were expected to achieve the same quality of work while deprived of the opportunity to be closer with and more available for patients.

Noticing various available affordances, such as the nearby forest and the in-house gym, but not being able to facilitate the use of these for all patients was not only a frustrating scenario for staff but also a major disappointment. Despite having relocated to a top-rank, modern, purposefully designed, and costly building, staff were unable to maximize the use of the facilities when it comes to available affordances in patient treatment, but also for staff’s experience of having a space to retreat and take a pause. Notably, a qualitative systematic review and meta-synthesis identified systemic and organizational difficulties as the most significant factors contributing to burnout (Vivolo et al., 2024). Furthermore, based on Vivolo et al.’s study clinical recommendations and implications for care included the support and improvement of workplace cultures in which staff well-being is protected.

Working in psychiatric care means being attentive and emotionally available to meet patients’ needs. Although sometimes stressful and demanding, this profession may also carry a sense of purpose and meaningfulness (Verderber, 2018). The opportunities and barriers to quality care shape health care practitioners therapeutic approaches and repertoire. In addition, as a work environment, the physical environment should facilitate their resilience and ability to cope in their daily work. A key factor in therapeutic work is the emotional and mental availability of staff to meet patients’ requirements, needs, and wishes. In this line of work, the relationship between performance and well-being among workers is dependent on the existence of breaks throughout a shift (Lyubykh et al., 2022). Additionally, calm places to which to withdraw to recover throughout their working day (Shepley et al., 2016) are essential for staff. In this study, the staff pointed to the lack of a designated, secluded place where they could recharge their batteries.

In many ways, the new building signals the prioritization of patients, where its resemblance to a hotel was thought to indicate a dignified place for providing mental health care. However, the staff gave many examples of the shortcomings of the universal design of the building; some aspects did not have the intended effect or align with patients’ needs. This was particularly true for elderly patients with cognitive impairments, for patients requiring longer stays, and for those who needed to be accompanied to make use of the facilities outside their respective wards.

Psychology as a field has been criticized for treating environments as mere backdrops to human activities (Clark & Uzzell, 2006). However, the last decade especially has seen an increased focus on how nature can provide support and co-facilitate therapy (Naor & Mayseless, 2021). By failing to acknowledge and be aware of the potential affordances for mental, physical, and relational well-being to be found in nature, psychology may be missing out on opportunities to promote health, healing, and recovery (Fernee et al., 2023). Our previous study noted expectations that the new mental health facility would offer a supportive and nature-inspired building design, which would add new dimensions to therapeutic practices (Hagerup et al., 2024).

The analytical results emphasized the potentially positive effects of nature, but the participants also reflected on daily barriers to seeking out nature. On the one hand, the design of the new mental health facility prioritized nature, recognizing its well-known benefits for physical and mental health. On the other hand, according to staff, the organizational changes in conjunction with the relocation hindered the frequent use of nature beyond the atrium gardens. While wild nature was considered to have a better effect on mental health, nevertheless, the building’s facilitation of a free flow between the indoor—outdoor spaces and access to daylight and natural scenery through the large windows were thought to reduce levels of aggression and stress. From the staff’s perspective, psychiatric staff are vulnerable to emotional fatigue, making the restorative effects of nature particularly valuable. Exposure to nature and natural environments has been shown to counteract compassion fatigue and mental tiredness, providing therapists and staff with opportunities for rejuvenation, creativity, and vitality (Jimenez et al., 2021; Larsen, 2020). A review of the role of nature in emotion regulation showed that exposure to nature appears to have a positive impact on emotion regulation processes and strategies (Vitale & Bonaiuto, 2024). These include reduced worrying and rumination, as well as improved adaptive strategies, such as mindfulness and cognitive reappraisal. Vitale and Bonaiuto (2024) further concluded an interplay between nature connectedness and affect regulation processes, which they suggested mediates the effects of contact with nature on perceived stress and happiness. In the context of the present study, nature as a mediator could offer important benefits for patients and staff alike.

In summary, we identified frustration and fatigue on the part of staff when numerous available affordances offered by a carefully designed health care building could not be realized due to organizational hindrances and time constraints. Furthermore, it is problematic for a modern facility that fosters a supportive environment for patients to be unable to offer such spaces for staff. This study also pointed to the limitations of a universal design, which may not necessarily meet all patient needs given the variability in diagnoses, treatment durations, impairments, and/or security concerns. These findings could inform future design processes to prevent the occurrence of similar mismatches.

## Methodological strengths and limitations

This phenomenological case study provided insights into the meanings of the physical environment based on staff’s experiences of therapeutic practice in a new mental health facility. We could have conducted individual interviews to gather in-depth knowledge, but we chose the focus group approach to investigate collective perspectives and voices. Focus group interviews may generate a wider range of ideas, as well as reveal aspects of group dynamics. Focus groups are criticized for the potential influence of the moderator. However, the researcher effect is also present in individual interviews and in other stages of the research process. Therefore, quality assurance measures need to be implemented regardless of the data generation method (Wibeck, 2010). In this study, there was no professional relationship between the participants and the moderator; however, they did share similar cultural and professional backgrounds, which is often perceived as an advantage to research (Wibeck, 2010). The focus group interviews were conducted only a few months after the staff and patients had relocated to the new building. Reinterviewing staff may have added a further dimension and could have potentially led to the discovery of differences in experiences after the building had been in use for longer, when initial problems might no longer have been relevant.

Phenomenology reveals meaning and reflectivity by sensing the world of things, others, and self, according to van Manen (2014). This means that conducting and writing up phenomenological research not only involves heads and hands but our whole sensual and sentient beings. Being aware of this, we found it challenging to find the right level of abstraction in the analysis and to process the large amounts of data generated by the three focus group interviews.

Although the interviews generated a considerable amount of data, the time available for carrying out each interview was a clear limitation, as we were restricted to the time the staff were at work. Because the participants were on duty, they could only be absent from their units for an hour, which left us little time to settle the participants in the room, to tune them in to the group and the topic of inquiry, and to conduct the actual interview. Although this study provided some insights into the inpatient care and therapeutic work of psychiatric staff in a new mental health facility, it was nevertheless a case study within a specific context. As such, the results and observations are not directly transferable to any other psychiatric hospital.

## Conclusions

In this study, we applied the theory of affordances and a phenomenological approach in the analysis of three focus group interviews. The objectives were to understand how staff relate to the physical environment in a new and purposefully designed mental health facility and to identify possibilities and hindrances to utilizing the affordances identified in the building itself and its natural surroundings. One conclusion concerns the importance, when designing new mental health care facilities, of analysing how the workflow and content of care will change. Psychiatric staff leverage their own skills and capacities for work as tools in patient care, remaining attentive to meeting patients’ practical and emotional needs, which is a demanding task. Given the character and emotional dimensions of psychiatric work, it is essential to provide staff with sufficient rest spaces, as their well-being directly influences the quality of care. In this context, it appears that contact with nature offers respite, the opportunity to clear the mind, and rejuvenation for staff and patients alike.
