# Correction

**DOI:** 10.1080/17482631.2025.2489263

**Published:** 2025-04-08

**Article title**: Psychiatric spaces: a phenomenological case study of staff perspectives after relocation to a new mental health facility

**Authors**: Anne Hagerup, Carina Ribe Fernee, Helle Wijk, Göran Lindahl and Sepideh Olausson

**Journal**: *International Journal of Qualitative Studies on Health and Well-being*

**Bibliometrics**: Volume 20, Number 01, pages 1-13

**DOI**: https://doi.org/10.1080/17482631.2025.2485697

The authors have noted that the name of the photographer, Marcel Tiedje, was omitted in the published article. The phrase “Photographs by Marcel Tiedje” has now been added beneath all the figures. This correction does not affect the description, interpretation, or the original conclusions of the article. The authors apologize for any inconvenience caused.

